# Determination of the Strength of Adhesion between Lipid Vesicles

**DOI:** 10.1100/2012/146804

**Published:** 2012-01-04

**Authors:** Tomáš Mareš, Matej Daniel, Aleš Iglič, Veronika Kralj-Iglič, Miha Fošnarič

**Affiliations:** ^1^Laboratory of Biomechanics, Faculty of Mechanical Engineering, CTU in Prague, Technická 4, 166 07 Prague 6, Czech Republic; ^2^Laboratory of Biophysics, Faculty of Electrical Engineering, University of Ljubljana, Tržaška 25, 1000 Ljubljana, Slovenia; ^3^Laboratory of Clinical Biophysics, Faculty of Medicine, University of Ljubljana, Lipičeva 2, 1000 Ljubljana, Slovenia

## Abstract

A commonly used method to determine the strength of adhesion between adhering lipid vesicles is measuring their effective contact angle from experimental images. The aim of this paper is to estimate the interobserver variations in vesicles effective contact angle measurements and to propose a new method for estimating the strength of membrane vesicle adhesion. Theoretical model shows for the old and for the new measure a monotonic dependence on the strength of adhesion. Results obtained by both measuring techniques show statistically significant correlation and high interobserver reliability for both methods. Therefore the conventional method of measuring the effective contact angle gives qualitatively relevant results as the measure of the lipid vesicle adhesion. However, the new measuring technique provides a lower variation of the measured values than the conventional measures using the effective contact angle. Moreover, obtaining the adhesion angle can be automatized more easily than obtaining the effective contact angle.

## 1. Introduction

Adhesion of membranes is ubiquitous in biological, biochemical, and biophysical processes. Therefore many theoretical models and experimental techniques have been developed that use or study membrane adhesion [1–4], an important segment of which is the adhesion of closed vesicles [5–7]. For example, adhesion of a vesicle represents an essential step for efficient drug delivery by small vesicles [8] and for many processes in biological cells, such as endo- and exocytosis or fusion of cells [9].

The range of interactions responsible for membrane adhesion is of the order of several nanometers [5]. If the size of the vesicle is much larger, typically of the order of a micrometer, we can simplify the adhesion potential by an effective contact energy [5]. The energy gain due to adhesion is then simply proportional to the contact area. In experimental practice, a commonly used method to extract the strength of adhesion from experimental images of the two adhering lipid vesicles is measuring the effective contact angle between the adhering vesicles. For example, this method was used to assess the capability of solution to mediate attractive interaction between membranous structures [10–13], since it was suggested that this mechanism suppresses vesiculation by causing the buds to adhere to the mother vesicle [14]. Shedding of microvesicles from cell membranes is an important process in cells [15] leading to cell-cell communication and thereby spreading of tumor and inflammation [16]. Since adhesion of buds to the mother membrane may suppress microvesiculation in vivo, mediated attractive interaction between membranous structures is a possible anticoagulant, anti-metastatic and anti-inflamatory effect [17].

However, the effective contact angle measurements are in practice difficult to perform fully objectively, and we are not aware of any analysis—theoretical or experimental—of the validity and the range of the “subjectivity” error of the above measuring technique.

The aim of this paper is to estimate the interobserver variations in effective contact angle measurements and to propose a new method for estimating the strength of membrane vesicle adhesion. The method is developed for phase contrast microscopy images of the two adhering lipid vesicles, but can be applied to other experimental techniques where contour of vesicles can be obtained or the proposed measure can be extracted by other means. In contrast to the conventional method of measuring adhesion through the effective contact angle, the measure proposed in this paper can be automatized more easily.

Both methods are discussed within the frames of a simple theoretical model and tested and compared in practice on a set of independent observers. The results of the statistical analysis of the data and its implications on the validity of both experimental techniques are discussed.

## 2. Theoretical Model

Let us discuss the experimental methods for determination of the strength of adhesion between two lipid vesicles in the frames of a simple theoretical model. Both vesicles have the same bending rigidities of their membranes, the same membrane areas, and the same vesicle volumes. The vesicles are adhered to each other and are rotationally symmetric around the axis normal to the adhesion plane.

The overall free energy (*F*) of the system of two adhering vesicles is taken as the sum of the bending energies (*W*_b_) of both membranes of the vesicles and an additional energy gain due to adhesion,

(1) $$F=W_{\text{b},1}+W_{\text{b},2}-\gamma A_{\text{adh}},$$

where *γ* is the adhesion constant and *A*_adh_ is the contact area. For the bending energy of the *i*th vesicle we use a Helfrich expression for zero spontaneous curvature [18]:

(2) $$W_{\text{b},i}=\frac{\kappa }{2}\overset{}{\underset{A_{i}}{\int }}{\left(C_{1}+C_{2}\right)}^{2}\text{d}A_{i},$$

where *κ* is the bending constant of the lipid membrane, *C*_1_ and *C*_2_ are the principle curvatures, and the integral runs over the membrane surface of vesicle *i* = 1,2. The Gaussian contribution [18] to the bending energy is omitted since it is constant for the nonchanging topology of our case.

We introduce the dimensionless *reduced adhesion constant*, $\tilde{\gamma }=\gamma A/\kappa$, where *A* = *A*_1_ = *A*_2_ is the area of each vesicle. Equation (1) in the dimensionless form is

(3) $$f=w_{\text{b},1}+w_{\text{b},2}-\tilde{\gamma }a,$$

where *f* = *F*/*κ*, *w*_b,*i*_ = *W*_b,*i*_/*κ* and *a* = *A*_adh_/*A*.

**Figure 1:**
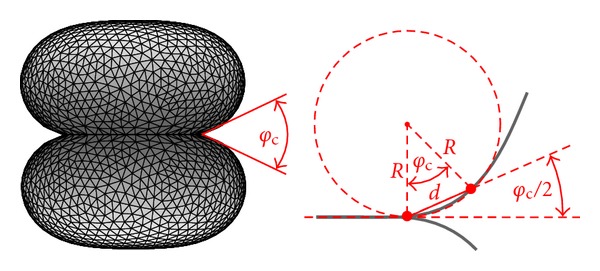
Left: a stable state of the two adhering vesicles obtained by minimization of the energy (3) for the relative volume of each vesicle *v* = 0.9 and for the dimensionless reduced adhesion constant $\tilde{\gamma }=10$. The effective contact angle *φ*_c_ is defined in the right panel. Right: definition of the effective contact angle. The membrane (shown in gray), has at the contact point the contact curvature 1/*R*. The secant that defines the effective contact angle *φ*_c_ cuts the membrane at the point of contact and at the distance *d* that should be small, that is, *d* ≪ *R*.

The stable configuration of the adhered vesicles is obtained as a shape that minimizes the free energy *f* in (3) for a constant area (*A* = const.) and volume (*V*_1_ = *V*_2_ = *V* = const.) of each vesicle. The stable configuration depends on the two parameters only: the dimensionless reduced adhesion constant $\tilde{\gamma }$ and the relative volume of each vesicle, $v=6\sqrt{\pi }V/A^{3/2}$. For illustration, Figure 1 (left) shows a stable state for *v* = 0.9 and $\tilde{\gamma }=10$.

In the case of no adhesion, that is, $\tilde{\gamma }\to 0$, the vesicles are independent and the contact area tends to zero. In the limit of strong adhesion, that is, $\tilde{\gamma }\to \infty$, the scale invariant bending energy becomes negligible compared with adhesion energy. In this limit, the shape of each vesicle approaches the shape of a spherical cap.

## 3. Effective Contact Angle

In the frames of the model described above, the membrane at the contact points, that is, at the rim of the contact area of the vesicles, has one principal curvature zero and the other one—the contact curvature—is determined by the relation [19, 20],

(4) $$\frac{1}{R}=\sqrt{\frac{2\gamma }{\kappa }},$$

where *R* is the radius of contact curvature.

Now let us consider the plane of the contact curvature, that is, the cross-sectional plane of the adhered vesicles that is normal to the contact plane and includes the symmetry axis. In this plane we define a secant that cuts the membrane at the contact point and at some distance *d* from the contact point. For small enough distances, *d* ≪ *R*, where the membrane has a shape of an arc of a circle with radius *R*, we can define an *effective contact angle* (see Figure 1),

(5) $$\varphi_{\text{c}}=2\arcsin\left(\frac{d}{2R}\right).$$

Note that in the limit of vanishing distance *d*, the above defined secant becomes a tangent to the membrane in the contact point. Then the effective contact angle tends to zero if the membrane bending energy is not negligible compared with the adhesion energy, or, equivalently, if the vesicles are not very large [5]. However, one can still use the effective contact angle for measuring adhesion also at moderate adhesion strengths. Using the relation for contact curvature from (4) and using it in the above definition of the effective contact angle, we can express the adhesion constant as

(6) $$\gamma =\frac{2\kappa }{d^{2}}\sin^{2}\left(\frac{\varphi_{\text{c}}}{2}\right).$$

This relation was the basis for estimating the strength of adhesion between lipid vesicles from confocal microscope images [11, 12].

In (6), the adhesion constant *γ* depends on the choice of the distance *d*. Since this distance has to be small, *d* ≪ *R*, the value of *γ* is in principle difficult to obtain. In practice, one usually draws on the microscope image two secants that appear as tangents to the membrane at the point of contact (see Figure 1). Although with this method the value of *d* is not precisely known, it assures that *d* ≪ *R*. And, using the same length scale for different images, it also assures a relatively small variation of *d* for all images. Nevertheless, how to draw the secants remains a subjective decision of the observer.

## 4. Adhesion Angle

**Figure 2:**
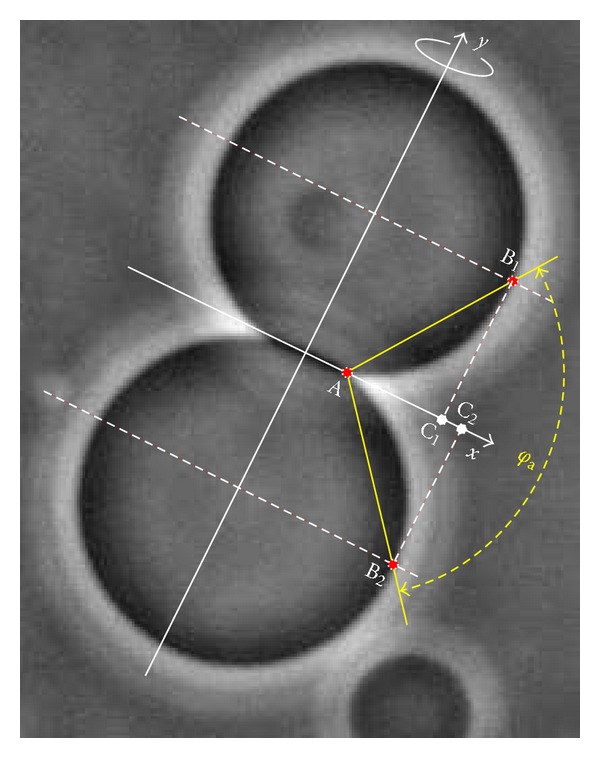
Phase contrast microscope image of the two adhering giant phospholipid vesicles (with radii of approx. 30 *μ*m). The *y*-axis denotes the symmetry axis and the *x*-axis the contact plane. Points A, B_1_, and B_2_ are to be located to measure the adhesion angle *φ*_a_ between lines $\bar{\text{A}{\text{B}}_{1}}$ and $\bar{\text{A}{\text{B}}_{2}}$. The adhesion angle can be obtained also from measuring lengths: *φ*_a_ = arctan*M*_1_ + arctan*M*_2_, where $M_{1}=\bar{{\text{B}}_{1}{\text{C}}_{1}}/\bar{\text{A}{\text{C}}_{1}}$, $M_{2}=\bar{{\text{B}}_{2}{\text{C}}_{2}}/\bar{\text{A}{\text{C}}_{2}}$, and points *C*_1_ and *C*_2_ are projections of points *B*_1_ and *B*_2_ to the contact plane.

In order to reduce the role of magnification and subjective factors in experimental determination of the adhesion constant *γ*, we propose in this work a new measure for the strength of adhesion between two lipid vesicles that can be easily obtained from images where contours of the vesicles can be identified, for example, from the phase contrast microscope images. We propose the following procedure (see Figure 2):

1. on the image locate the axis of the rotational symmetry of the two vesicle system (denoted on Figure 2 as the *y*-axis),
2. on the image locate points A; B_1_, and B_2_: point A lays in the contact plane (denoted on Figure 2 as the *x*-axis) where the membranes of the two vesicles separate; points B_1_ and B_2_ are where the membranes are the farthest away from the symmetry axis *y*;
3. measure the *adhesion angle φ*_a_ as an angle between lines $\bar{\text{A}{\text{B}}_{1}}$ and $\bar{\text{A}{\text{B}}_{2}}$.

The proposed adhesion angle is relatively simple to obtain, for example by using the ImageJ software [21], where angles can be measured directly and interactively on the image. One can, however, instead of measuring the angle, measure the distances $\bar{\text{A}{\text{B}}_{1,2}}$ and $\bar{{\text{B}}_{1,2}{\text{C}}_{1,2}}$ as defined in Figure 2. By calculating measures $M_{1}=\bar{{\text{B}}_{1}{\text{C}}_{1}}/\bar{\text{A}{\text{C}}_{1}}$ and $M_{2}=\bar{{\text{B}}_{2}{\text{C}}_{2}}/\bar{\text{A}{\text{C}}_{2}}$, we can get the adhesion angle from *φ*_a_ = arctan*M*_1_ + arctan*M*_2_.

Note that for a given value of the dimensionless reduced adhesion constant $\tilde{\gamma }=\gamma A/\kappa$, the measure is scale invariant and depends only on the relative volume of the vesicles. Therefore, one only needs to obtain the angle of adhesion and the relative volume of the vesicles to extract $\tilde{\gamma }$. The relative volume of the vesicle can be obtained, for example, by extracting the contour of the vesicle from the image through some automated image segmentation method. Then the volume and the area of the vesicle with the rotational symmetry around the *y*-axis are *V* = *π*∫*x* ^2^d*y* and *A* = 2*π*∫*x*d*l*, where *x* is the distance of the membrane from the *y*-axis and $\text{d}l=\sqrt{\text{d}x^{2}+\text{d}y^{2}}=\sqrt{1+{\left(\text{d}x/\text{d}y\right)}^{2}}\text{d}y$. The relative volume is, as defined in Section 2, $v=6\sqrt{\pi }V/A^{3/2}$.

Let us show that the new proposed measure of adhesion is a monotonically increasing function of the reduced adhesion constant $\tilde{\gamma }$ (see (3)) in the frames of a simple theoretical model described in Section 2. There we have a symmetry across the contact plane. Then the adhesion angle can be written as *φ*_a_ = arctan*M*_1_ + arctan*M*_2_ = 2arctan*M*, where due to the symmetry *M*_1_ = *M*_2_. In the following we will see that *M* = tan(*φ*_a_/2) is a monotonic function of the reduced adhesion constant $\tilde{\gamma }$, which means that also the adhesion angle itself is a monotonic function of $\tilde{\gamma }$.

**Figure 3:**
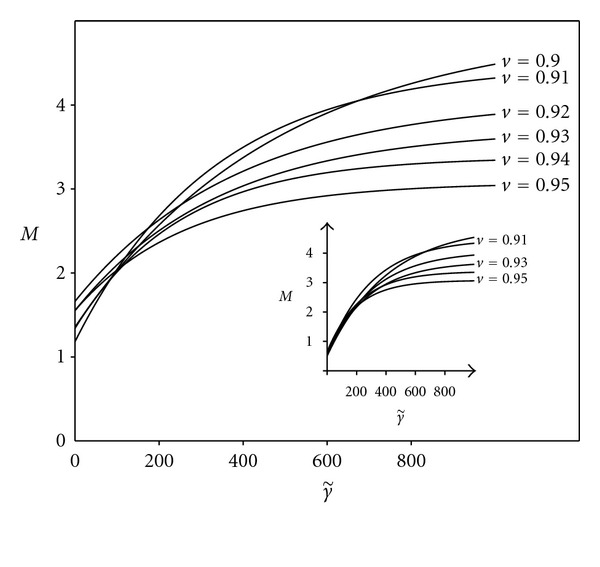
Measure *M* = tan(*φ*_a_/2), where *φ*_a_ is the adhesion angle, as a function of dimensionless reduced adhesion constant $\tilde{\gamma }$ for the vesicle relative volumes *v* = 0.9–0.95. Curves show the fits of function from (7) where only *k* is a free parameter. The inset shows the fit, where the constraint *M*_0_ is released, and the data is fitted using the function defined in (7) for the two free parameters: *M*_0_ and *k*.

Figure 3 shows the dependence of *M* = tan⁡(*φ*_a_/2) on the dimensionless reduced adhesion constant for different values of the relative volume *v*. The figure was obtained by minimization of the dimensionless overall free energy of the system of the two adhering vesicles (3). The minimization was performed using the Surface Evolver software [22], and the data was then fitted using the least square method on the function

(7) $$M=\left(M_{\infty }-M_{0}\right)\left(1-e^{-k\tilde{\gamma }}\right)+M_{0},$$

where $M_{0}=\lim_{\tilde{\gamma }\to 0}M$ and $M_{\infty }=\lim_{\tilde{\gamma }\to \infty }M$.

In Figure 3, the curves are a fit using (7) where *M*_0_ is obtained from the case of zero tension, and *M*_*∞*_ from the strong adhesion limit, where the bending energy becomes irrelevant compared with adhesion energy and the shape of each vesicle approaches the shape of the spherical cap (see page 92 in [5]). Then the adhesion angle depends only on the relative volume *v* of the vesicle through a geometric relation

(8) $$\begin{array}{c} v=\frac{4+6\sin\varphi_{\text{a}}-2\;\sin^{3}\varphi_{\text{a}}}{{\left[2+2\sin\varphi_{\text{a}}+\cos^{2}\varphi_{\text{a}}\right]}^{3/2}}. \end{array}$$

Therefore, only *k* is a free parameter for fitting the curve in (7). For illustration, the inset shows the fit where the constraint *M*_0_ is released, and the data is fitted using the function defined in (7) for the two free parameters: *M*_0_ and *k*.

Note also that in the strong adhesion limit the vesicle obtains a shape of a spherical cap. Then the effective contact angle is nonzero also for *d* = 0 and can be defined independently of *d* as *φ*_c_^(0)^ = lim⁡_*d*→0_ *φ*_c_. The geometry of a spherical cap yields a relation between the adhesion angle and the effective contact angle in the limit of strong adhesion: *φ*_a_ + *φ*_c_^(0)^/2 = *π*/2.

The proposed measure of adhesion is applicable to vesicles of all sizes above a few hundreds of nanometers in diameter. This limit is set by the fact that the range of interactions responsible for membrane adhesion (several nanometers) has to be much smaller than the size of the vesicle, so that the adhesion potential can be simplified by an effective contact energy [5].

## 5. Measuring Techniques in Practice

Determination of the strength of adhesion between the vesicles was tested in practice for a conventional technique using the effective contact angle and for the technique introduced in this work that uses the adhesion angle.

For that purpose, the effective contact angle and the adhesion angle between two lipid vesicles were measured by five independent observers on a set of 100 phase contrast microscope images. Giant phospholipid vesicles with various lipid compositions were prepared and recorded using an inverted microscope with phase-contrast optics, as in details explained elsewhere [12]. Only images with two adhering vesicle were selected, with vesicles various in size (10 *μ*m–100 *μ*m) and relative volume. All observers were involved in the studies of cell mechanics and were familiar with the vesicle adhesion measurements. The effective contact angle and the adhesion angle were measured independently on both sides of the vesicles in the image plane. measurements were performed using the ImageJ software [21].

Each observer provided 200 measurements for each type of the measured angle. Intraclass correlation coefficients (ICC) were calculated as a measure of interobserver reliability [23]. The ICC takes values between 0 and 1 and is close to 1 when the differences between paired measurements are very small compared to the differences between subjects. ICC was calculated according to Shrout and Fleiss schema [24].

Values of ICC in Table 1 indicate that both methods are significantly reliable with respect to the interobserver variability. However, the adhesion angle has statistically significant higher (*P* < 0.05) interobserver reliability in comparison to the conventional measurements of the effective contact angle.

**Figure 4:**
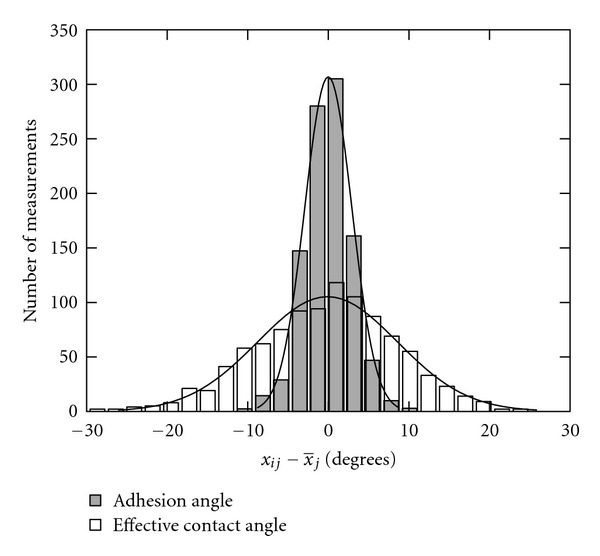
A histogram of the differences between the measured angles, *x*_*ij*_, measured on the sample of *j*_max⁡_ = 200 intervesicle contacts by *i*_max⁡_ = 5 raters and the average value of all raters for the given angle, ${\bar{x}}_{j}$. Empty (white) bars represent measurements of the effective contact angle, the conventional measure of adhesion, and solid (gray) bars represent the measures of the adhesion angle, the new measure of adhesion proposed in this work. Solid lines represent best fit of the normal distribution.

As is shown in Figure 4, the adhesion angle measurement technique provides considerable lower variations in values measured by different observers than the effective contact angle measurement technique.

**Figure 5:**
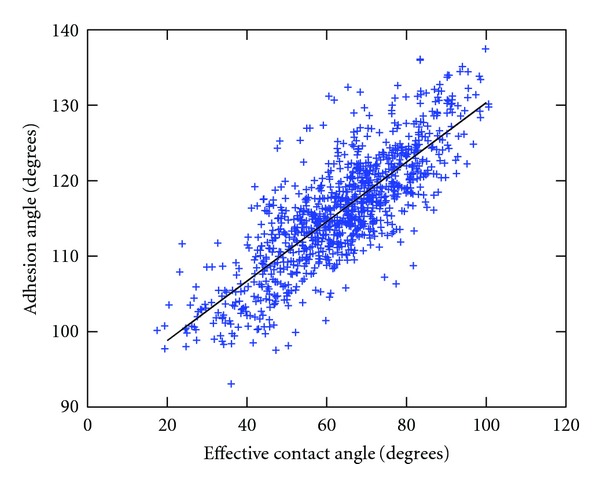
Correlation between the measured values of the effective contact angle (conventional method for measuring adhesion) and the adhesion angle (here proposed measure of adhesion).

Figure 5 shows that new proposed method is consistent with the conventional measurements of the effective contact angle, as can be seen from significant correlation between the values obtained by both methods (Pearson correlation coefficient 0.80791, *P* < 0.001, 95% confidence interval 0.78076 to 0.83202).

Based on the definition, the new proposed measure of adhesion provides for the adhesion angle higher values then the conventional method for the effective contact angle.

## 6. Conclusion

Adhesion of biological and lipid vesicle membranes is an important biological, biochemical, and biophysical process. In experimental practice, one possible method to extract the strength of adhesion from experimental images of the two adhering lipid vesicles is measuring the effective contact angle between the adhering vesicles.

However, there are drawbacks measuring the effective contact angle, since the secants of the membrane that define the effective contact angle have to be very close to the tangents to the membrane at the contact point. How to draw this secants remains a subjective decision of the observer and depends on the length scale of the image. For the above reasons, measuring of the effective contact angle is not easily amendable for automation.

Therefore we proposed in this work a new—simple and objective—method to extract the strength of adhesion from experimental images of two adhering lipid vesicles. The method is developed for images obtained by phase contrast microscopy, but can be applied to other experimental techniques where contour of vesicles can be obtained or the proposed measure can be extracted by other means.

The new measure was tested within a simple theoretical model of the two identical adhering vesicles. The model yields the monotonic dependence of the proposed measure on the reduced adhesion constant and is therefore an appropriate measure for the strength of adhesion.

Both methods—the new and the conventional one—were also tested in practice on a set of independent observers using the same set of phase contrast microscope images. Statistical analysis of the measured data shows significant correlation between the methods and high interobserver reliability of both methods. Therefore, we can conclude that the conventional method of measuring the effective contact angle gives qualitatively relevant results. However, the new measuring technique provides a lower variation of the measured values than the conventional measures using the effective contact angle. And, obtaining the adhesion angle can be automatized more easily than the effective contact angle.

## Figures and Tables

**Table 1:** Intraclass correlation coefficient (ICC) as a measure of interobserver reliability. The *P* values and the 95% confidence intervals for both types of the measured angles are also shown.

Method	ICC	*P*	95% conf. interval
Eff. contact angle (*φ*_1_)	0.7620	*P* < 0.001	0.7182 to 0.8028
Adhesion angle (*φ*_2_)	0.8440	*P* < 0.001	0.8126 to 0.8725
